# Playing a Musical Instrument as a Protective Factor against Dementia and Cognitive Impairment: A Population-Based Twin Study

**DOI:** 10.1155/2014/836748

**Published:** 2014-12-02

**Authors:** M. Alison Balbag, Nancy L. Pedersen, Margaret Gatz

**Affiliations:** ^1^Davis School of Gerontology, University of Southern California, 3715 McClintock Avenue, Los Angeles, CA 90089-0191, USA; ^2^Department of Psychology, University of Southern California, Los Angeles, CA 90089-1061, USA; ^3^Department of Medical Epidemiology and Biostatistics, Karolinska Institutet, 171 77 Stockholm, Sweden

## Abstract

Increasing evidence supports that playing a musical instrument may benefit cognitive development and health at young ages. Whether playing an instrument provides protection against dementia has not been established. In a population-based cotwin control study, we examined the association between playing a musical instrument and whether or not the twins developed dementia or cognitive impairment. Participation in playing an instrument was taken from informant-based reports of twins' leisure activities. Dementia diagnoses were based on a complete clinical workup using standard diagnostic criteria. Among 157 twin pairs discordant for dementia and cognitive impairment, 27 pairs were discordant for playing an instrument. Controlling for sex, education, and physical activity, playing a musical instrument was significantly associated with less likelihood of dementia and cognitive impairment (odds ratio [OR] = 0.36 [95% confidence interval 0.13–0.99]). These findings support further consideration of music as a modifiable protective factor against dementia and cognitive impairment.

## 1. Introduction

As we face unprecedented population aging, there is a strong focus on identifying those factors that may promote healthy cognitive aging and protect against age-related neurodegenerative diseases, such as dementia. Although more attention has focused on the cognitive effects of playing a musical instrument in youth [1–5], this type of musical engagement holds interest as a protective factor in later life cognition and neurodegenerative diseases [6, 7]. However, the effect of playing an instrument as a leisure activity on dementia risk has not been thoroughly investigated. Some previous dementia studies have not explicitly included playing an instrument as a stand-alone activity but combined it with other activities in leisure lists: “knitting or music or other hobby” as one item [8], for example, or “practicing an artistic activity” as one item [9]. Studies that have explicitly listed playing an instrument in leisure lists have yielded mixed results: while one study [10] did find playing an instrument frequently to be protective against dementia, as opposed to rarely playing, another did not find a significant protective effect [11].

Because dementia is generally accepted to be caused by a combination or interaction of genetic and environmental influences throughout the life span, unmeasured confounding in traditional designs may produce biased results, as noted in previous work [10]. One important confound may be genetic propensity to not only the outcome of interest but also the exposure. Studying twins offers a unique solution to this methodological concern by significantly reducing confounding by genetic and familial sources [12, 13], thus allowing us to more accurately investigate how prior music engagement may be associated with cognitive health outcomes. This design improves on a traditional case control study in the following ways. (1) In addition to being matched on typical characteristics such as age or race, twins are by design matched in varying degrees on genetic propensity: monozygotic (MZ) twins share 100% of their genetic makeup and dizygotic (DZ) twins share on average 50%. (2) Because twins are typically reared together they are also matched on environmental exposures encountered during formative developmental years that may influence long-term health [12]. Previous twin studies examining leisure activities and dementia risk have not included playing a musical instrument as a focus of study [13, 14].

We investigated the effect of playing a musical instrument on dementia risk using a cotwin control design, where twin pairs discordant for a disease are studied to determine risk factors unique to the twin with the disease, as well as protective factors exclusive to the twin without the disease. We hypothesized that, in twin pairs where one member of the pair had developed dementia or cognitive impairment, the unaffected twin would be more likely to have played a musical instrument in older adulthood.

## 2. Methods

### 2.1. Participants

Data for the present study come from the Study of Dementia in Swedish Twins, known as HARMONY [15]. Case identification entailed a screening phase and clinical phase. All individuals from the population-based Swedish Twin Registry (STR) aged 65 years and older and alive at baseline were eligible for screening from 1998 to 2001 (*n* = 20,269 eligible). Participants were invited to the clinical phase if they scored poorly on cognitive screening and were considered a suspect for dementia. Twin partners to dementia suspects were also invited to participate in the clinical phase for cotwin analyses. A complete clinical workup including neuroimaging was used to assess dementia in clinical phase participants. In HARMONY, 1713 individuals participated in the clinical phase and were either given a clinical diagnosis or confirmed to be intact cognitively.

All participants' consent was obtained according to the Declaration of Helsinki and the study was approved by the Ethics Committee of the Karolinska Institute and the Institutional Review Board of the University of Southern California.

### 2.2. Measures

#### 2.2.1. Dementia

Clinical diagnostic procedures followed the Consortium to Establish a Registry for Alzheimer's Disease (CERAD) [16] and included neurological assessment of memory, language, and perceptual motor domains, physical and neurological examination, a complete medical history, informant interviews, and neuroimaging referral. A consensus clinical diagnosis was reached, with participants determined as meeting diagnostic criteria for dementia according to the* Diagnostic and Statistical Manual of Mental Disorders, 4th Edition* [17], having questionable dementia (i.e., meeting two of three criteria for clinical diagnosis of dementia), or being cognitively intact. In this report, we refer to the questionable group as cognitively impaired. Differential diagnosis of Alzheimer's disease used criteria from NINDS-ADRDA [18].

In the present study, cases included those with clinical diagnoses of dementia, as well as those with cognitive impairment. Controls included those diagnosed as cognitively intact. We required the controls to live at least until their cotwin's age of dementia onset or age at diagnosis of cognitive impairment. A sensitivity analysis for dementia cases alone was performed, omitting those with questionable dementia.

#### 2.2.2. Playing a Musical Instrument

In the clinical phase of HARMONY, additional data were collected on participants to help identify risk and protective factors for dementia [15]. Among the protocols was a leisure activities questionnaire based on the Florida Cognitive Activities Scale [19]. This information was obtained from informants of both demented and nondemented participants in order to have comparable data from cases and controls. Participants themselves also completed the questionnaire if they were cognitively able. In the present study, the informant-based reports were used for both cases and controls in order to minimize loss of data for those with dementia and reduce recall bias, reduce source bias, and provide a consistent method of assessment across cases and controls. The supplemental self-report was only used in the event where an informant-based report was not available for a participant. To support this strategy, an interrater reliability analysis was performed to determine consistency between the informant-based reports and self-reports of playing an instrument, and a sensitivity analysis was performed for informant reports alone.

In the questionnaire, respondents were asked about the participant's experience of playing a musical instrument, if any. Questions included whether the participant played new and/or familiar music, frequency of playing, and whether she/he was still playing at time of assessment or stopped playing at a specific age.

We included as musicians those participants who played new and/or familiar music frequently and/or occasionally at time of assessment. To minimize reverse causation and account for the fact that a lifelong player may have stopped playing because of dementia onset, we also deemed participants to be musicians if they stopped playing within five years prior to dementia onset. Controls were also required to play at least until five years of their cotwin's dementia onset to be included as a musician. In pairs where the case had cognitive impairment, age at clinical assessment was used in lieu of onset age for the aforementioned cutoff point. Sensitivity analyses were performed with wider than five-year exclusion points. In the present study, there were no professional musicians.

### 2.3. Cotwin Control Design

**Figure 1:**
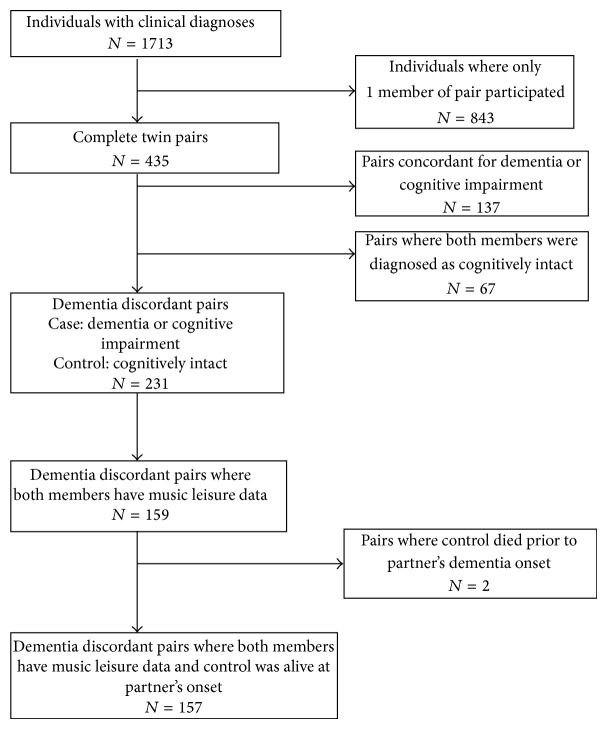
Flowchart illustrating how cotwin sample was obtained from HARMONY.

In HARMONY, 231 twin pairs were discordant for dementia or cognitive impairment and 137 twin pairs were concordant for dementia or cognitive impairment. After excluding pairs with missing music data or pairs where the control died before the case's age of onset, the present sample consisted of 157 twin pairs (*n* = 314 individuals) discordant for dementia or cognitive impairment.  Figure 1 illustrates how the current sample was obtained.

### 2.4. Covariates

Because the present study employed a cotwin control design, age was automatically controlled for within pairs. Additional variables controlled for included sex and two factors that the existing dementia literature suggests may be protective: education [20–24] and physical activity [25, 26]. Education was measured by years of education. Physical activity was assessed by two items included in the leisure activities questionnaire (walks (at least 30 minutes) and exercise (e.g., aerobics, jogging, golf, and tennis)) with a similar response format to the music items.

### 2.5. Statistical Analysis

Statistical analyses were performed using SAS statistical software version 9.2 [27]. Conditional logistic regression (SAS PROC LOGISTIC with the STRATA statement to stratify by each twin pair) was used for within-pair analyses and to estimate odds ratios (ORs) and corresponding 95% confidence intervals.

## 3. Results

### 3.1. Participant Characteristics

Characteristics of the 157 pairs are reported in Table 1. Among cases, 66% of participants were diagnosed with dementia and 34% with cognitive impairment. The majority of dementia cases were Alzheimer's disease.

### 3.2. Test of Hypothesis

Thirty-one individuals played an instrument as an older adult. Four pairs were concordant for music. In 17 pairs, the musician was cognitively intact. In 6 pairs, the musician was demented or cognitively impaired. Characteristics of the 27 pairs where at least one twin was a musician are reported in Table 2.

Our analyses confirm the hypothesis that twins who played a musical instrument in older adulthood were less likely to develop dementia and cognitive impairment compared to their cotwins. A crude test found playing an instrument to be significantly associated with less likelihood of dementia and cognitive impairment (OR = 0.35, 95% CI: 0.14–0.90). Controlling for sex, education, and physical activity, playing an instrument remained significantly associated with less likelihood of dementia and cognitive impairment (OR = 0.36, 95% CI: 0.13–0.99). In other words, compared to their nonmusician cotwin, musicians playing an instrument in older adulthood had a 64% lower likelihood of developing dementia or cognitive impairment.

### 3.3. Sensitivity Analyses

In 104 of the 157 pairs, the case was diagnosed with dementia. The association was similar among these pairs alone (crude OR = 0.36, 95% CI: 0.12–1.14), although nonsignificant due to a limited number of musicians within these pairs. Separate analyses by zygosity (crude OR = 0.5, 95% CI: 0.05–5.5 for MZ pairs; crude OR = 0.44, 95% CI: 0.14–1.44 for DZ pairs) found associations in the protective direction, although nonsignificant due to a limited number of pairs. Additionally, analyses of the 121 pairs where both twins had an informant report for music found a nearly identical association with the results including self-report data (crude OR = 0.39, 95% CI: 0.14–1.08; full model adjusting for sex, education, and physical activity OR = 0.36, 95% CI: 0.12–1.08). Analyses for these 121 pairs, however, were nonsignificant due to having fewer musicians within these pairs. Moreover, sensitivity analyses for exclusion points wider than five years found similar associations (crude OR = 0.38, 95% CI: 0.15–0.96 for seven years; crude OR = 0.5, 95% CI: 0.21–1.17 for ten years).

### 3.4. Consistency of Informant versus Self-Reports of Playing an Instrument

Among the 157 pairs, 13% of participants had only self-reports of playing a musical instrument, including 24 controls and 16 cases. Using 180 individuals for whom there were both complete informant reports and self-reports available, the interrater reliability between reporters was weighted Kappa = 0.72 (*P* < .0001), 95% CI: 0.59–0.84, for playing new music and weighted Kappa = 0.74 (*P* < .0001), 95% CI: 0.62–0.86, for playing familiar music. Moreover, there was neither greater nor lesser likelihood for self-reported engagement in music versus informant-reported engagement in music. These results support use of self-report when an informant-based report was not available.

## 4. Discussion

In this investigation of music's influence on cognitive health outcomes, the results from this cotwin control design find that playing an instrument in older adulthood is significantly associated with reduced likelihood of dementia and cognitive impairment. Despite sharing numerous genetic propensities and environmental exposures during formative developmental years, dissimilarities in music engagement were associated with differences in dementia occurrence within twin pairs. Moreover, the association is not explained by education or physical activity.

Using a cotwin control design improves upon previous studies that have included playing an instrument in leisure lists [10, 11] by controlling for a large number of genetic and environmental factors. Additionally, because HARMONY is a population-based sample, the concern of selection bias can be considerably minimized. Use of informant-based reports minimized loss of data for those with dementia, while keeping method of assessment consistent across cases and controls.

One question raised when studying musicians is whether those who select into music are systematically different from those who do not, whether in terms of innate brain differences or external environmental influences, such as education or parental socioeconomic status [7, 28, 29]. Given the cotwin design, our results support previous suggestions that differences observed between musicians and nonmusicians are likely due to music training not preexisting biological differences [3, 28, 29].

The present study cannot speak to causal mechanisms. However, it has been suggested that the cognitive benefits associated with musical ability may grant older musicians better maintained cognitive reserve [6] as has been discussed with respect to other cognitively stimulating activities [8, 10] or may provide compensatory abilities to mitigate age-related cognitive declines [30]. Music processing is unique in that it necessitates a wide array of brain regions and functions simultaneously throughout both hemispheres [31–35].

A few limitations of this study should be mentioned. First, the music data are retrospective. However, analyses based on informant reports for both members of the pair do not differ from using combined self-report and informant reports, and, furthermore, we found no tendency of more musicians identified through self-report. Second, the frequency measures of playing an instrument were not anchored to number of times per week. Nonetheless, within-pair analyses allow us to identify pairs discordant for playing an instrument, regardless of absolute time spent playing each week. Third, we included both dementia and questionable dementia diagnoses as cases; however, a sensitivity analysis of only dementia cases revealed a nearly equivalent effect. Fourth, both MZ and DZ twin pairs were included in this cotwin control design because there were a limited number of dementia-discordant MZ pairs. Therefore, although genetic differences were not wholly controlled for, the twin design still enables us to account for early life environmental factors beyond a traditional case control design. A sensitivity analysis did not find notable differences in association with dementia risk among MZ pairs versus same-sex DZ pairs. Finally, we do not have data indicating the age at which a participant started playing their instrument. It is most likely that individuals who play as older adults learned to play earlier in life and are lifelong musicians. The importance of early music exposure for long-term cognitive trajectories is supported by previous work that finds older adult musicians who begin at a young age and play for ten or more years over the life course exhibit enhanced cognitive abilities compared to nonmusicians [7]. However, if these musicians began playing as older adults, our results may indicate that playing an instrument has a positive influence on neuroplasticity regardless of what age one begins playing. This suggestion is supported by a previous study which found that after six months of piano lessons older adults experienced better working memory and executive functioning than controls [36].

In conclusion, our results support consideration of music's potential role as a nonpharmacological, noninvasive, and modifiable health behavior protective against dementia and cognitive impairment.

## Figures and Tables

**Table 1:** Sample characteristics (*n* = 157 pairs).

	Cases (*n* = 157)		Controls (*n* = 157)
Age (years)^1^	78 ± 6.1 (65–92)		77.9 ± 6.1 (65–92)
Women	89 (56.7)		82 (52.2)
Men	68 (43.3)		75 (47.8)
Education (years)	7.8 ± 2.6 (2–16)		8 ± 2.6 (0–16)
Physical activity^2^	1 ± 1 (0–3)		1.4 ± 1 (0–3)
Zygotic status			
Monozygotic		39 (24.8)	
Same-sex dizygotic		67 (42.7)	
Opposite sex dizygotic		49 (31.2)	
Indeterminate		2 (1.3)	
Clinical diagnoses			
Dementia	104 (66.2)		
Cognitive impairment	53 (33.8)		
Cognitively intact			157 (100)

Data are *n* (%) or means ± SD (range).

^1^Mean age excludes pairs where control was not assessed in person due to death (*n* = 7 pairs).

^2^Physical activity was assessed using a 0–3 scale, where 0 is no activity and 3 is frequent activity.

**Table 2:** Characteristics for pairs where at least one twin was a musician (*n* = 27 pairs^1^).

	Cases (*n* = 27)		Controls (*n* = 27)	Odds ratio (OR) and 95% CI for crude model
Age (years)	78 ± 6.1 (66–89)		78 ± 6.1 (66–89)	
Women	11 (40.7)		10 (37)	
Men	16 (59.3)		17 (63)	
Education (years)	8.9 ± 3.4 (6–16)		8.6 ± 3.4 (6–16)	OR = 0.96 (0.9, 1.1)
Physical activity^2^	0.9 ± 1 (0–3)		1.5 ± 1 (0–3)	OR = 0.6^*^ (0.5, 0.8)
Musicians	10 (37)		21 (78)	OR = 0.35^*^ (0.14, 0.9)
Zygotic status				
Monozygotic		3 (11.1)		
Same-sex dizygotic		14 (51.9)		
Opposite sex dizygotic		9 (33.3)		
Indeterminate		1 (3.7)		
Clinical diagnoses				
Dementia	16 (59.3)			
Cognitive impairment	11 (40.7)			
Cognitively intact			27 (100)	

Data are *n* (%) or means ± SD (range).

^1^There were 31 musicians in the sample. In 4 pairs, both twins were musicians.

^2^Physical activity was assessed using a 0–3 scale, where 0 is no activity and 3 is frequent activity.

^*^ *P* < 0.05.
